# Concordance and timing in recording cancer events in primary care, hospital and mortality records for patients with and without psoriasis: A population-based cohort study

**DOI:** 10.1371/journal.pone.0254661

**Published:** 2021-07-19

**Authors:** Alex M. Trafford, Rosa Parisi, Martin K. Rutter, Evangelos Kontopantelis, Christopher E. M. Griffiths, Darren M. Ashcroft, on behalf of the Global Psoriasis Atlas (GPA)

**Affiliations:** 1 Division of Pharmacy and Optometry, School of Health Sciences, Faculty of Biology, Medicine and Health, NIHR Manchester Biomedical Research Centre, University of Manchester, Manchester, United Kingdom; 2 Division of Population Health, Health Services Research and Primary Care, School of Health Sciences, Faculty of Biology, Medicine and Health, NIHR School of Primary Care Research, University of Manchester, Manchester, United Kingdom; 3 Division of Diabetes, Endocrinology and Gastroenterology, School of Medical Sciences, Faculty of Biology, Medicine and Health, Manchester Academic Health Science Centre (MAHSC), The University of Manchester, Manchester, United Kingdom; 4 Diabetes, Endocrinology and Metabolism Centre, Manchester University NHS Foundation Trust, MAHSC, Manchester, United Kingdom; 5 Dermatology Centre, Salford Royal NHS Foundation Trust, Faculty of Biology, Medicine and Health, NIHR Manchester Biomedical Research Centre, University of Manchester, Manchester, United Kingdom; University of Oxford, UNITED KINGDOM

## Abstract

**Background:**

The association between psoriasis and the risk of cancer has been investigated in numerous studies utilising electronic health records (EHRs), with conflicting results in the extent of the association.

**Objectives:**

To assess concordance and timing of cancer recording between primary care, hospital and death registration data for people with and without psoriasis.

**Methods:**

Cohort studies delineated using primary care EHRs from the Clinical Practice Research Datalink (CPRD) GOLD and Aurum databases, with linkage to hospital episode statistics (HES), Office for National Statistics (ONS) mortality data and indices of multiple deprivation (IMD). People with psoriasis were matched to those without psoriasis by age, sex and general practice. Cancer recording between databases was investigated by proportion concordant, that being the presence of cancer record in both source and comparator datasets. Delay in recording cancer diagnoses between CPRD and HES records and predictors of discordance were also assessed.

**Results:**

58,904 people with psoriasis and 350,592 comparison patients were included using CPRD GOLD; whereas 213,400 people with psoriasis and 1,268,998 comparison patients were included in CPRD Aurum. For all cancer records (excluding keratinocyte), concordance between CPRD and HES was greater than 80%. Concordance for same-site cancer records was markedly lower (<68% GOLD-linked data; <72% Aurum-linked data). Concordance of non-Hodgkin lymphoma and liver cancer recording between CPRD and HES was lower for people with psoriasis compared to those without.

**Conclusions:**

Concordance between CPRD and HES is poor when restricted to cancers of the same site, with greater discordance in people with psoriasis for some cancers of specific sites. The use of linked patient-level data is an important step in reducing misclassification of cancer outcomes in epidemiological studies using routinely collected electronic health records.

## Introduction

Psoriasis is an immune-mediated inflammatory disease, with substantial regional variation in prevalence across the globe [1]. In Western Europe, the prevalence is relatively high when compared to many other global regions, with UK-specific estimates that it affects 2.8% of the general population [2]. The importance of psoriasis has been highlighted by the World Health Organization (WHO) [3], which acknowledged not only the burden of the disease to the individual and to society, but also the consequence of associated comorbidities. These comorbidities include psoriatic arthritis [4], cardiovascular disease [5], depression [6] and cancer [7]. As in other diseases, many recent studies investigating comorbid conditions in psoriasis have been conducted by using routinely collected electronic health records (EHRs). A recent systematic review [7] identified 37 studies examining the risk of cancer development in people with psoriasis conducted using such databases, with many only utilising primary care [8–11] or hospital [12–15] data. However, estimates quantifying the degree of risk remain varied. Several explanations have been posited for this variation, including the extent of adjustment for confounding and differing severities of psoriasis in study groups [16]. Whilst these explanations may play a role, it is also important to consider, given the proportion of studies conducted using EHRs, that some of the variation may come as a result of bias in the ascertainment of cancer outcomes.

Population-based EHR databases present a number of distinct advantages for epidemiological research, including increased power and consideration of multiple exposures [17], and increasingly are being used to address questions related to dermato-epidemiology. Given that such databases are derived from routinely recorded EHRs, it is important to consider the potential for misclassification of outcomes. Recently, a cohort study using the Clinical Practice Research Datalink (CPRD) found that the use of linked primary care and hospitalisation records helped to avoid outcome misclassification, as the use of primary care data alone to ascertain hospitalisation for lower respiratory tract infection would have underestimated the incidence rate by 31% [18]. Given that a number of studies have investigated the risk of cancer occurrence in psoriasis using only primary or secondary care data, it is important to consider the extent to which misclassification may be a problem. The primary aim of this study was therefore to assess the concordance of cancer recording between primary care, hospital, and national mortality records for people with and without psoriasis. Our secondary aim assessed the delay in recording between the different data sources for same-site cancer records and risk factors influencing discordance in recording.

## Materials and methods

### Data sources

#### Clinical Practice Research Datalink (CPRD) GOLD and Aurum

The CPRD is a UK-based research service consisting of primary care records and formed from two databases: GOLD and Aurum [19, 20]. Diagnostic information is recorded through Read codes, while information on test results, ethnicity and lifestyle measures (e.g. smoking status) is also available. Data from registered patients in the contributing general practices in England may be linked to a number of other data sources [21], including hospital records, in the form of Hospital Episode Statistics (HES) and mortality data, in the form of Office for National Statistics (ONS) death registration data. Socioeconomic data is also available for linkage through the Index of Multiple Deprivation 2010 (IMD), which is a small-area measure based on patient residential postcode, providing an aggregate measure of deprivation across seven domains, including income, employment and health [22].

#### Hospital Episode Statistics (HES)

Hospital episode statistics consist of records from inpatient, outpatient and emergency admissions in English NHS hospitals. Following patient discharge, clinicians complete a discharge summary that is then entered into an electronic patient information database. HES records contain a range of information, including patient demographics (including ethnicity), medical diagnoses at discharge (coded using ICD-10) and procedures (coded using OPCS-4) [23].

#### Office for National Statistics death registrations (ONS)

ONS mortality records are derived from death registrations. Upon patient death, an attending doctor completes a Medical Certificate of Cause of Death (MCCD) that is then passed on to a local registrar of births and deaths. Causes of death listed as part of a patients’ MCCD may be a primary or underlying cause, and are coded according to ICD-9 (pre-2001) or ICD-10 (2001-present) [24].

### Study population

Psoriasis patients were identified by a diagnostic Read code for psoriasis in the primary care records within the study period (01/01/1998–30/11/2018). Patients were required to be eligible for linkage to HES, ONS and IMD; be a minimum of 18 years old; and, have been in an ‘up to standard’ practice (those with continuity in reporting of data and expected death rates) for at least 12 months prior to study entry. Patients were excluded if they had any record of cancer (excluding keratinocyte cancer) prior to study entry in order to ensure only primary cancers were included. Additionally, patients with any diagnostic record of HIV/Aids prior to study entry were excluded due to the associated increased cancer risk. A bridging file was applied to the GOLD cohort to exclude patients and practices that transferred from CPRD GOLD to Aurum. Cohort delineation is presented in S1 Fig. The index date for psoriasis patients was defined as the first record of psoriasis in the study period.

Comparison patients were matched to psoriasis patients at a ratio of up to 6:1 on age, sex and general practice. Restriction criteria for comparison patients were consistent with those for psoriasis patients, with the addition of having no psoriasis record prior to study entry. Pseudo-index dates for comparison patients were generated based on the index date of their matched psoriasis patient. Psoriasis and comparison cohorts were identified separately for CPRD GOLD and Aurum. All patients were followed from index date to the first occurrence of cancer diagnosis, transfer out from the general practice, last data collection date, study period end or death.

### Data analysis

#### Outcomes

The primary outcome of interest was cancer diagnosis. In order to examine variation in concordance, cancer diagnoses were split into the following categories: all cancer (excluding keratinocyte cancer), bladder, brain, breast, cervical, colorectal, gallbladder, Hodgkin’s lymphoma (HL), keratinocyte, kidney, laryngeal, leukaemia, liver, lung, malignant melanoma, multiple myeloma, nasal cavity, non-Hodgkin’s lymphoma (NHL), oesophageal, oral cavity, ovarian, pancreatic, pharyngeal, prostate, stomach, thyroid and uterine cancer. Code lists were formed by one author (AMT) and were then separately reviewed by two clinicians (CEG and MKR), with any discrepancies rectified through discussion between both clinicians and AMT. Code lists for exposure and outcome are available for download from www.clinicalcodes.org [25]. Data analysis was carried out using Stata version 16 (Statacorp, College Station, TX, USA).

#### Assessment of concordance

Concordance of cancer recording was considered between one source database and a comparison database (i.e. CPRD as the source database with HES as the comparison database, and vice versa). Records in the comparison database must have occurred prior to transfer out date, last data collection date, study period end or death in order to be eligible for consideration of concordance. Concordance between the source database and the comparison database was classified into 3 groups: (1) same site recorded in the source and comparison database (2) any cancer recorded in the comparison database (3) no record in the comparison database. Additionally, for each cancer record in the source database, it was evaluated whether there was any death registration including a record of cancer. Factors associated with discordance in reporting between the source and comparison databases, for CPRD and HES, were assessed using logistic regression. The following risk factors for discordance were included in the model: age, gender, deprivation and time period. Where the same cancer-site record was found in the comparison database, the delay in recording between the source and comparison records was examined.

This study was approved by the Independent Scientific Advisory Committee (ISAC) for Medicines and Healthcare Regulatory Agency database research (ISAC approval 19_089R).

## Results

In CPRD GOLD, 58,904 people with psoriasis and 350,592 matched comparison patients were included. In CPRD Aurum, 213,400 people with psoriasis and 1,268,998 matched comparison patients were included (Table 1).

**Table 1:** Demographic characteristics of psoriasis and comparison cohorts.

	CPRD GOLD		CPRD Aurum	
	Psoriasis cohort	Comparison cohort	Psoriasis cohort	Comparison cohort
No.	58904	350592	213400	1268998
Female (%)	30301 (51.4)	180108 (51.4)	108539 (50.9)	644,353 (50.8)
Age, median (IQR)	47.2 (33.9–61.5)	47.1 (33.9–61.3)	45.9 (33.0–60.5)	45.7 (32.9–60.3)
Indices of Multiple Deprivation (%)				
1 (least deprived)	13266 (22.5)	81276 (23.2)	46454 (21.8)	286172 (22.6)
2	12591 (21.4)	76874 (21.9)	44660 (20.9)	270343 (21.3)
3	12186 (20.7)	72400 (20.7)	41290 (19.4)	242733 (19.1)
4	11235 (19.1)	64829 (18.5)	41608 (19.5)	244115 (19.2)
5 (most deprived)	9595 (16.3)	55055 (15.7)	39191 (18.4)	224354 (17.7)
missing	31 (0.1)	158 (0.1)	197 (0.1)	1281 (0.1)
Ethnic group				
White	49698 (84.4)	269071 (76.8)	171759 (80.5)	920950 (72.6)
Asian	1296 (2.2)	7971 (2.3)	7949 (3.7)	50370 (4.0)
Black	272 (0.5)	4789 (1.4)	1630 (0.8)	28255 (2.2)
Other	594 (1.0)	4529 (1.3)	2297 (1.0)	16705 (1.3)
Unknown	7044 (12.0)	64232 (18.0)	29765 (14.0)	252718 (20.0)

### Concordance in cancer recording

**Fig 1:**
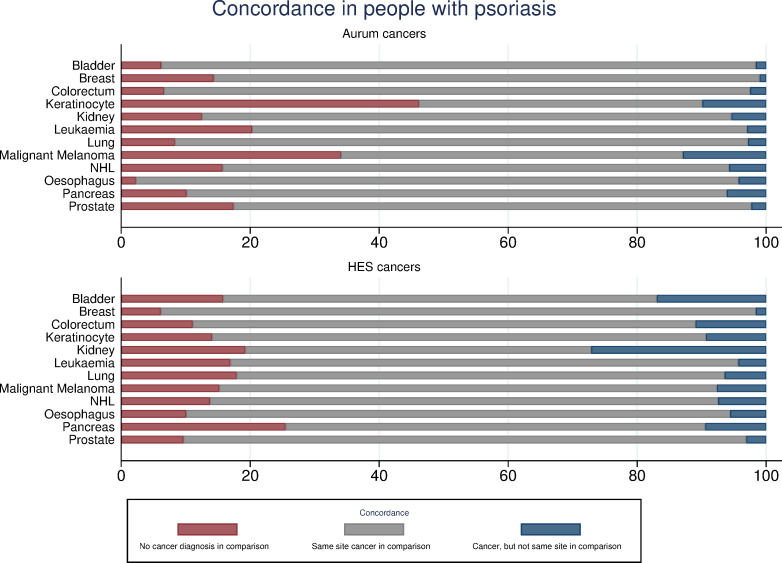
Concordance in cancer recording for Aurum-linked people with psoriasis.

**Fig 2:**
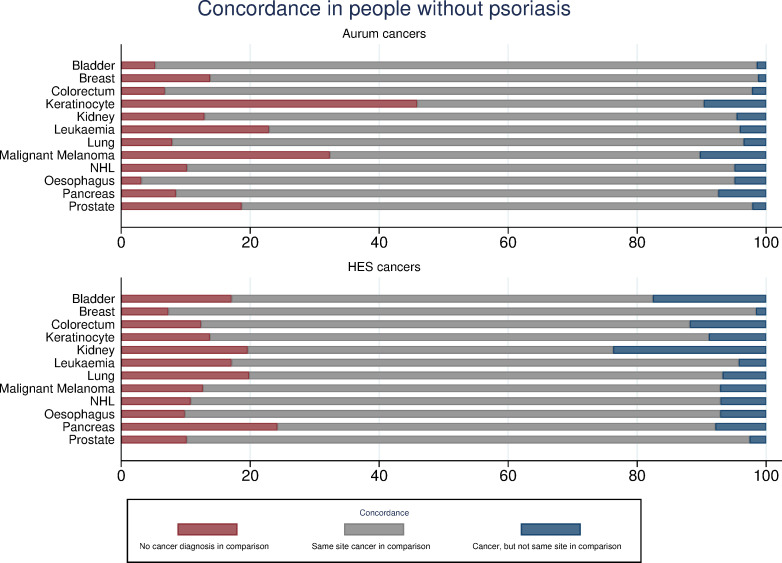
Concordance in cancer recording for Aurum-linked comparison patients.

The concordance of cancer recording between CPRD Aurum, HES and ONS is reported separately for both people with psoriasis (Fig 1) and comparison patients (Fig 2). Concordance for CPRD GOLD-linked data is reported in S2 and S3 Figs.

#### CPRD-identified cancer records

In CPRD Aurum, 11,889 and 63,691 cancers (excluding keratinocyte) were identified in psoriasis and comparison patients respectively. Concordance at the same site in HES was 71.0% for people with psoriasis and 71.5% for comparison patients. Concordance for any record of cancer was higher (84.5% psoriasis; 84.8% comparison). In CPRD GOLD, 2,916 and 15,236 cancers events (excluding keratinocyte) were identified in the psoriasis and comparison group, respectively. Same cancer-site concordance with HES was 67.9% for people with psoriasis and 67.7% for comparisons. Concordance of any cancer record (excluding keratinocyte) was higher (82.7% psoriasis; 82.2% comparison). When examining specific cancers, concordance between CPRD identified records and HES was lowest for keratinocyte cancers and malignant melanoma in both CPRD Aurum and GOLD. In people with psoriasis, for any cancer (excluding keratinocyte) record in CPRD, 35.2% (Aurum) and 35.3% (GOLD) also had a cancer record in ONS mortality records.

#### HES-identified cancer records

In CPRD Aurum-linked HES, 11,777 and 63,298 cancers (excluding keratinocyte) were identified; 70.9% and 71.2% of these had a cancer record at the same site in CPRD Aurum for people with psoriasis and comparisons respectively. Any site record concordance was higher (84.0% psoriasis; 84.2% comparison). In patients with psoriasis 2,911 cancers (excluding keratinocyte) were identified in CPRD GOLD-linked HES and 15,124 cancers were identified in comparison patients. Of these, 67.3% (psoriasis) and 67.8% (comparison) had a record at the same site in CPRD GOLD. Any cancer record concordance for CPRD GOLD was 81.6% (psoriasis) and 81.6% (comparison). For site-specific cancers, concordance between HES identified cancers and CPRD records was lowest for pancreatic, lung and kidney cancer. In people with psoriasis, for any cancer (excluding keratinocyte) record in HES, 40.7% (CPRD Aurum-linked HES) and 40.8% (CPRD GOLD-linked HES) also had a cancer record in ONS mortality records.

#### Variation in concordance by psoriasis status

When considering same cancer site concordance between source and comparator databases, notable differences were found between psoriasis and comparison patients for two cancers. Of the liver cancers recorded in CPRD Aurum-linked HES for people with psoriasis, 28% had no record in CPRD Aurum. In comparison, for the psoriasis-free patients, 20% of liver cancer records were only found in HES. For non-Hodgkin lymphoma (NHL), a greater proportion of cases were only found in CPRD Aurum for people with psoriasis (16%) compared to comparison patients (10%). CPRD GOLD results followed the same pattern and are included in the (S3 and S4 Tables).

### Delays in recording cancer events between sources

**Fig 3:**
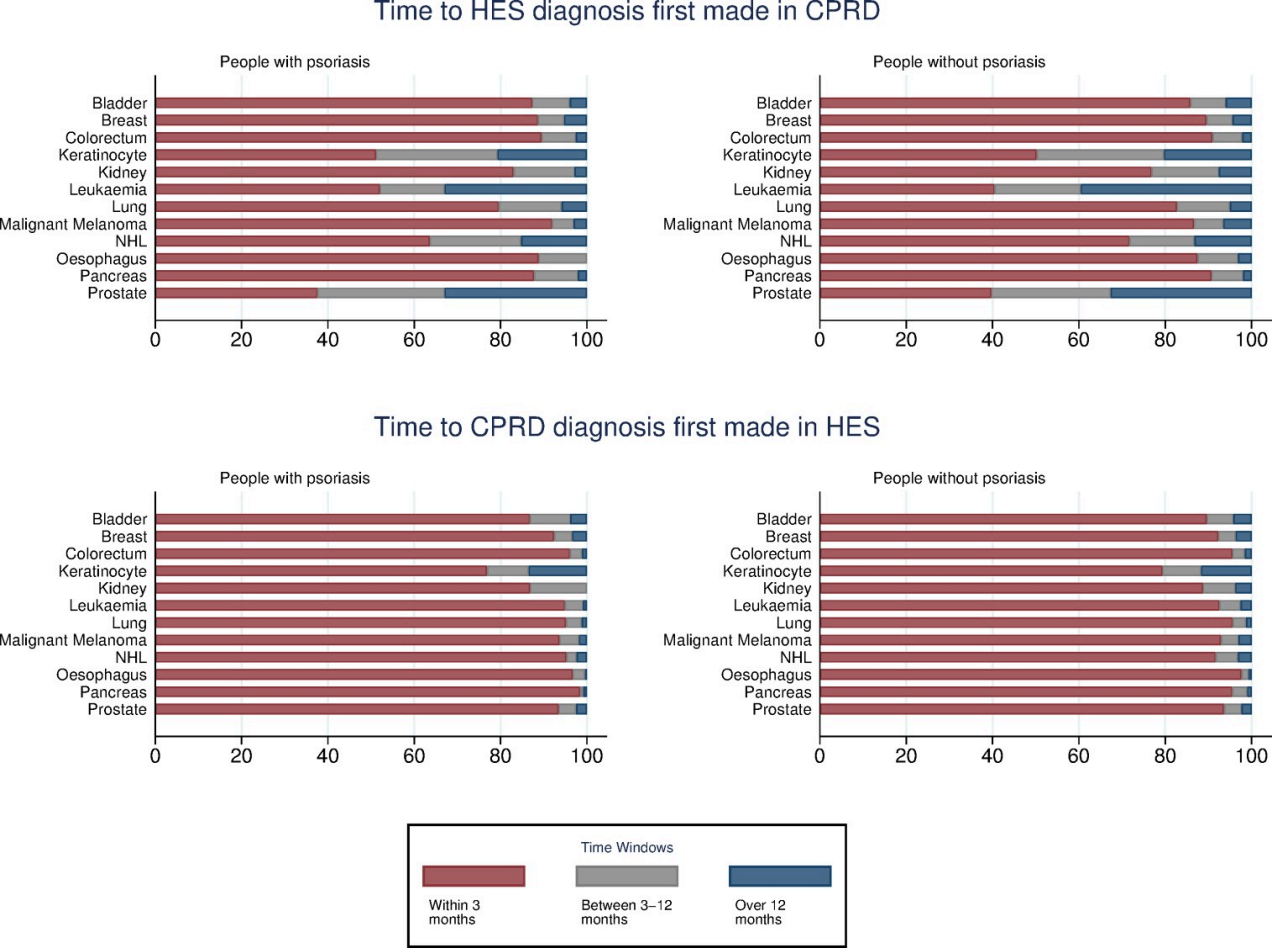
Delay between same site concordant record by cancer site for Aurum-linked data.

There was little variation in the timing of recording of cancer events between people with psoriasis and comparison patients or between CPRD GOLD-linked data and CPRD Aurum-linked data (Table 2). For records first identified in CPRD, between 75–79% were identified within 3 months in HES. The large majority of records first identified in HES were recorded in CPRD within 3 months (>90%), with less than 4% having a delay of over a year. There was notable variation in the delay between records by cancer site, particularly for records first identified in the CPRD. In records first identified in CPRD Aurum, the lowest proportion of concordant records identified within three months was observed for keratinocyte cancers, leukaemia, non-Hodgkin lymphoma and prostate cancer (Fig 3). Variation in delay for records first identified in HES was less apparent, with only keratinocyte cancers having a notably lower proportion of concordant records within 3 months. Results for the CPRD GOLD cohort were similar and are included in the (S4 Fig).

**Table 2:** Delay between same site cancer records in source and comparator database.

Delay to record in comparison data	CPRD GOLD				CPRD Aurum			
	Psoriasis		Comparison		Psoriasis		Comparison	
	CPRD first record	HES first record	CPRD first record	HES first record	CPRD first record	HES first record	CPRD first record	HES first record
Within 3 months	859 (76.83)	941 (92.53)	4504 (78.37)	5121 (93.93)	3791(77.07)	3997 (94.18)	20516 (76.95)	21387 (94.15)
3 to 12 months	135 (12.08)	41 (4.03)	668 (11.62)	205 (3.76)	626 (12.73)	181 (4.26)	3285 (12.32)	872 (3.84)
Over year	124 (11.09)	35 (3.44)	575 (10.01)	126 (2.31)	502 (10.21)	66 (1.56)	2859 (10.72)	456 (2.01)
Total	1118	1017	5747	5452	4919	4244	26660	22715

### Risk factors for discordance in cancer recording

In CPRD Aurum, increased age was associated with discordance in cancer recording when either CPRD or linked HES data was the source database, regardless of whether patients had psoriasis (Table 3). Compared to those under 64, those aged 65–74 (OR 1.29, 95%CI: 1.23–1.36) and over 75 (OR 2.06, 95%CI: 1.95–2.18) were more likely to have a record that only appeared in HES. Only those aged over 75 were more likely than those aged less than 65 to only have a record in CPRD Aurum (OR 1.43, 95%CI: 1.35–1.51). For both people with psoriasis and comparison patients, the odds of having a record only in the primary care records reduced with increasing deprivation. In contrast, where HES was the source database, discordance was more likely in the comparison cohort for those in more deprived areas. With regards to temporality, later year of diagnosis was also associated with reduced discordance. Factors associated with discordance in the CPRD GOLD cohort were similar and are presented in the (S5 Table).

**Table 3:** Multivariable-adjusted odds of non-concordance according to covariates in CPRD-Aurum and linked HES data.

	Psoriasis—Aurum Source			Psoriasis—HES Source			Comparison—Aurum Source			Comparison—HES Source		
	Odds Ratio	95% CI		Odds Ratio	95% CI		Odds Ratio	95% CI		Odds Ratio	95% CI	

Age categories												
Under 64	1.00	reference		1.00	reference		1.00	reference		1.00	reference	
65 to 74	0.93	0.83	1.05	1.22	1.09	1.37	1.01	0.96	1.07	1.29	1.23	1.36
Over 75	1.38	1.21	1.58	1.80	1.59	2.05	1.43	1.35	1.51	2.06	1.95	2.18
Gender												
Male	1.00	reference		1.00	reference		1.00	reference		1.00	reference	
Female	1.09	0.98	1.20	1.06	0.96	1.18	1.02	0.98	1.07	1.04	1.00	1.09
Deprivation												
1 (least deprived)	1.00	reference		1.00	reference		1.00	reference		1.00	reference	
2	0.92	0.80	1.06	0.83	0.72	0.96	0.88	0.83	0.94	0.98	0.91	1.04
3	0.79	0.68	0.92	0.82	0.70	0.96	0.81	0.76	0.86	1.10	1.03	1.17
4	0.75	0.65	0.88	1.07	0.92	1.24	0.73	0.68	0.78	1.17	1.09	1.25
5 (most deprived)	0.63	0.53	0.75	1.03	0.88	1.20	0.63	0.59	0.68	1.29	1.20	1.38
Period												
1998/2000	1.00	reference		1.00	reference		1.00	reference		1.00	reference	
2001/2003	0.71	0.50	1.00	0.86	0.59	1.26	0.75	0.63	0.89	0.80	0.68	0.94
2004/2006	0.40	0.29	0.56	0.52	0.36	0.75	0.63	0.54	0.75	0.56	0.48	0.65
2007/2009	0.34	0.25	0.47	0.57	0.40	0.80	0.55	0.47	0.65	0.51	0.44	0.60
2010/2012	0.36	0.27	0.49	0.58	0.41	0.82	0.55	0.47	0.64	0.47	0.40	0.55
2013/2015	0.38	0.28	0.52	0.57	0.41	0.80	0.60	0.51	0.70	0.49	0.42	0.57
2016/2018	0.57	0.43	0.77	0.60	0.43	0.85	0.84	0.72	0.99	0.57	0.49	0.66

## Discussion

This study examined the concordance of cancer recording between primary care, hospital and death registration data in people with and without psoriasis. Concordance of cancer records at the same site between CPRD and HES was poor, with marked variation according to cancer site. Though higher, concordance for any cancer record remained below 85%. The delay between same-site records varied according to the database in which the cancer was first identified, with older age, time period and deprivation associated with discordance in reporting.

Concordance for cancer records of the same site differed according to the cancer site in question and to the database in which the cancer was first identified. For cancers initially identified in the CPRD, same-site concordance in HES was notably lower for keratinocyte cancers and malignant melanoma. Conversely, for cancers initially identified in HES, same-site concordance in the CPRD was lowest for pancreatic, lung and kidney cancers. Previously suggested plausible explanations for lower same-site concordance include the use of non-specific cancer diagnostic codes, death shortly following hospital admission for cancer, and death from cancer prior to hospitalisation [26], with the latter two explanations supported by increased likelihood of discordance in older patients. With regards to keratinocyte cancers specifically, high discordance between CPRD and HES records likely arises as appropriately trained primary care physicians are able to excise lesions without referral to secondary care [27]. As lower concordance of same-site records suggests a potential for poor outcome ascertainment in studies utilising only one data source, these results support the need to link primary and secondary care data sources in CPRD studies of cancer occurrence, especially those considering site-specific cancers.

Differences in concordance between people with and without psoriasis were present for some site-specific cancers, with implications for studies considering associations between psoriasis and cancer. For NHL identified in HES, site-specific concordance within CPRD was lower for people with psoriasis compared to those without. As cutaneous T-cell lymphoma (CTCL), a variant of NHL, clinically manifests in a manner which mimics psoriasis [28], it is possible that people with CTCL are misdiagnosed as having psoriasis in primary care. Referral to specialist hospital services would likely result in an accurate CTCL diagnosis and a consequential discordant cancer record. Furthermore, in people with psoriasis, CPRD identified NHL records were also more commonly discordant–likely resulting from patients receiving an incorrect CTCL diagnosis in primary care, which is then correctly identified as psoriasis in secondary care. The link between psoriasis and lymphoma has received significant focus in previous work [10, 11, 29] and these results suggest caution in the interpretation of these findings. Studies utilising only primary or secondary care records that do not differentiate between NHL variants are likely to over-estimate the risk of NHL through the aforementioned misclassification of CTCL cases in psoriasis patients.

There was also heterogeneity in the recording of liver cancer cases between people with and without psoriasis, with a greater proportion of liver cancer cases only identified in HES for people with psoriasis. It is plausible that this discordance arises as psoriasis patients have higher liver cancer mortality and therefore die before a record is made in the CPRD–an argument supported by increased heavy drinking in people with psoriasis [30] and the suggested increased mortality in alcohol-associated liver cancer [31]. Under this explanation, studies that only use primary care records may underestimate the association between psoriasis and liver cancer.

Beyond psoriasis, age, deprivation and time period were all predictors of discordance. As noted previously, increasing age was associated with an increased probability of having a discordant record–likely resulting from cancer death prior to a record being made in the comparison data source. For records first identified in the CPRD, increasing deprivation was associated with lower discordance. Rather than suggesting improved recording practices in more deprived areas, it is likely that this relationship is explained by the increased incidence of the most commonly discordant cancers, such as keratinocyte cancers [32], malignant melanoma [33] and prostate cancer [34], in less deprived areas. With regards to time, reduced discordance in later periods is suggestive of improved data recording, as noted for lifestyle and demographic factors in previous works [20].

To our knowledge, this is the first study to consider the concordance of cancer recording between CPRD Aurum and linked HES data, and the inclusion of both GOLD and Aurum within the study allows cross-validation between the two databases. A potential limitation, as in any study of concordance between EHRs, are discrepancies in coding dictionaries. However, code list review by multiple clinicians was carried out to minimise any issues.

In conclusion, the use of primary care or hospital data in isolation to determine cancer events is likely to be inadequate, particularly when considering certain site-specific cancers. In addition, these inadequacies may be exacerbated through improper consideration of important predictors of discordance. As such, the use of linked electronic health records, with appropriate covariate consideration, is strongly advisable in studies of cancer risk as a means of improving outcome ascertainment.

## Supporting information

S1 Fig Diagram of participant selection process. (DOCX)

S2 Fig Concordance in cancer recording for GOLD-linked people with psoriasis. (TIF)

S3 Fig Concordance in cancer recording for GOLD-linked comparison patients. (TIF)

S4 Fig Delay between same site concordant record by cancer site for GOLD-linked data. (TIF)

S1 Table Concordance in cancer recording for Aurum-linked people with psoriasis. (DOCX)

S2 Table Concordance in cancer recording for Aurum-linked comparison patients. (DOCX)

S3 Table Concordance in cancer recording for GOLD-linked people with psoriasis. (DOCX)

S4 Table Concordance in cancer recording for GOLD-linked comparison patients. (DOCX)

S5 Table Multivariable-adjusted odds of non-concordance according to covariates in CPRD-GOLD linked data. (DOCX)

## References

[1] Parisi R, Iskandar IYK, Kontopantelis E, Augustin M, Griffiths CEM, Ashcroft DM. National, regional, and worldwide epidemiology of psoriasis: systematic analysis and modelling study. BMJ. 2020;369:m1590. doi: 10.1136/bmj.m1590 32467098 PMC7254147

[2] Springate DA, Parisi R, Kontopantelis E, Reeves D, Griffiths CE, Ashcroft DM. Incidence, prevalence and mortality of patients with psoriasis: a U.K. population-based cohort study. Br J Dermatol. 2017;176(3):650–8. doi: 10.1111/bjd.15021 27579733 PMC5363241

[3] World Health Organization. Global report on psoriasis. World Health Organisation. 2016. Available from: https://apps.who.int/iris/handle/10665/204417

[4] Gelfand JM, Gladman DD, Mease PJ, Smith N, Margolis DJ, Nijsten T, et al. Epidemiology of psoriatic arthritis in the population of the United States. J Am Acad Dermatol. 2005;53(4):573.e1–.e13. doi: 10.1016/j.jaad.2005.03.046 16198775

[5] Miller IM, Ellervik C, Yazdanyar S, Jemec GB. Meta-analysis of psoriasis, cardiovascular disease, and associated risk factors. J Am Acad Dermatol. 2013;69(6):1014–24. doi: 10.1016/j.jaad.2013.06.053 24238156

[6] Dowlatshahi EA, Wakkee M, Arends LR, Nijsten T. The prevalence and odds of depressive symptoms and clinical depression in psoriasis patients: a systematic review and meta-analysis. J Invest Dermatol. 2014;134(6):1542–51. doi: 10.1038/jid.2013.508 24284419

[7] Trafford AM, Parisi R, Kontopantelis E, Griffiths CEM, Ashcroft DM. Association of Psoriasis With the Risk of Developing or Dying of Cancer: A Systematic Review and Meta-analysis. JAMA Dermatol. 2019;155(12):1390–403. doi: 10.1001/jamadermatol.2019.3056 31617868 PMC6802036

[8] Brauchli YB, Jick SS, Miret M, Meier CR. Psoriasis and risk of incident cancer: an inception cohort study with a nested case-control analysis. J Invest Dermatol. 2009;129(11):2604–12. doi: 10.1038/jid.2009.113 19440219

[9] Chiesa Fuxench ZC, Shin DB, Ogdie Beatty A, Gelfand JM. The Risk of Cancer in Patients With Psoriasis: A Population-Based Cohort Study in the Health Improvement Network. JAMA Dermatology. 2016;152(3):282–90. doi: 10.1001/jamadermatol.2015.4847 26676102 PMC5273859

[10] Gelfand JM, Berlin J, Van Voorhecs A, Margolis DJ. Lymphoma rates are low but increased in patients with psoriasis—Results from a population-based cohort study in the United Kingdom. Arch Dermatol. 2003;139(11):1425–9. doi: 10.1001/archderm.139.11.1425 14623702

[11] Gelfand JM, Shin DB, Neimann AL, Wang X, Margolis DJ, Troxel AB. The risk of lymphoma in patients with psoriasis. J Invest Dermatol. 2006;126(10):2194–201. doi: 10.1038/sj.jid.5700410 16741509

[12] Chen YJ, Wu CY, Chen TJ, Shen JL, Chu SY, Wang CB, et al. The risk of cancer in patients with psoriasis: a population-based cohort study in Taiwan. J Am Acad Dermatol. 2011;65(1):84–91. doi: 10.1016/j.jaad.2010.04.046 21458106

[13] Chiou MJ, Fang YF, Kuo CF. Increased risk of cancer in patients with psoriasis: A nationwide population study. Ann Rheum Dis. 2016;75:606.

[14] Egeberg A, Thyssen JP, Gislason GH, Skov L. Skin cancer in patients with psoriasis. Journal of the European Academy of Dermatology and Venereology. 2016; 30: 1349–53. doi: 10.1111/jdv.13619 26932589

[15] Frentz G, Olsen JH. Malignant tumours and psoriasis: a follow-up study. Br J Dermatol. 1999;140:237–42. doi: 10.1046/j.1365-2133.1999.02655.x 10233215

[16] Pouplard C, Brenaut E, Horreau C, Barnetche T, Misery L, Richard MA, et al. Risk of cancer in psoriasis: a systematic review and meta-analysis of epidemiological studies. J Eur Acad Dermatol Venereol. 2013;27:36–46. doi: 10.1111/jdv.12165 23845151

[17] Casey JA, Schwartz BS, Stewart WF, Adler NE. Using Electronic Health Records for Population Health Research: A Review of Methods and Applications. Annu Rev Public Health. 2016;37(1):61–81. doi: 10.1146/annurev-publhealth-032315-021353 26667605 PMC6724703

[18] Yiu ZZN, Parisi R, Lunt M, Warren RB, Griffiths CEM, Langan SM, et al. Risk of hospitalization and death due to infection in people with psoriasis: a population-based cohort study using the Clinical Practice Research Datalink. Br J Dermatol. 2020;184(1):78–86. doi: 10.1111/bjd.19052 32222069

[19] Wolf A, Dedman D, Campbell J, Booth H, Lunn D, Chapman J, et al. Data resource profile: Clinical Practice Research Datalink (CPRD) Aurum. Int J Epidemiol. 2019;48(6):1740-g. doi: 10.1093/ije/dyz034 30859197 PMC6929522

[20] Herrett E, Gallagher AM, Bhaskaran K, Forbes H, Mathur R, van Staa T, et al. Data Resource Profile: Clinical Practice Research Datalink (CPRD). Int J Epidemiol. 2015;44(3):827–36. doi: 10.1093/ije/dyv098 26050254 PMC4521131

[21] Padmanabhan S, Carty L, Cameron E, Ghosh RE, Williams R, Strongman H. Approach to record linkage of primary care data from Clinical Practice Research Datalink to other health-related patient data: overview and implications. Eur J Epidemiol. 2019;34(1):91–9. doi: 10.1007/s10654-018-0442-4 30219957 PMC6325980

[22] Noble M, Wright G, Smith G, Dibben C. Measuring Multiple Deprivation at the Small-Area Level. Environment and Planning A: Economy and Space. 2006;38(1):169–85.

[23] Herbert A, Wijlaars L, Zylbersztejn A, Cromwell D, Hardelid P. Data Resource Profile: Hospital Episode Statistics Admitted Patient Care (HES APC). Int J Epidemiol. 2017;46(4):1093–i. doi: 10.1093/ije/dyx015 28338941 PMC5837677

[24] Office for National Statistics. User guide to mortality statistics. Office for National Statistics. 2019. Available from: https://www.ons.gov.uk/peoplepopulationandcommunity/birthsdeathsandmarriages/deaths/methodologies/userguidetomortalitystatisticsjuly2017.

[25] Springate DA, Kontopantelis E, Ashcroft DM, Olier I, Parisi R, Chamapiwa E, et al. ClinicalCodes: An Online Clinical Codes Repository to Improve the Validity and Reproducibility of Research Using Electronic Medical Records. PLOS ONE. 2014;9(6):e99825. doi: 10.1371/journal.pone.0099825 24941260 PMC4062485

[26] Williams R, van Staa T-P, Gallagher AM, Hammad T, Leufkens HGM, de Vries F. Cancer recording in patients with and without type 2 diabetes in the Clinical Practice Research Datalink primary care data and linked hospital admission data: a cohort study. BMJ Open. 2018;8(5): e020827. doi: 10.1136/bmjopen-2017-020827 29804063 PMC5988054

[27] National Institute for Health and Care Excellence (NICE). Improving outcomes for people with skin tumours including melanoma: evidence update October 2011. NICE. 2011. Available from: https://www.nice.org.uk/guidance/csg8/evidence/evidence-update-october-2011-pdf-2188923229 31891468

[28] Martinez-Escala ME, Posligua AL, Wickless H, Rutherford A, Sable KA, Rubio-Gonzalez B, et al. Progression of undiagnosed cutaneous lymphoma after anti-tumor necrosis factor-alpha therapy. J Am Acad Dermatol. 2018;78(6):1068–76. doi: 10.1016/j.jaad.2017.12.068 29307643 PMC5951749

[29] Kamstrup MR, Skov L, Zachariae C, Thyssen JP, Egeberg A. Psoriasis and risk of malignant lymphoma: a population-based cohort study. Br J Dermatol. 2018;178(6):1435–6. doi: 10.1111/bjd.16245 29247449

[30] Parisi R, Webb RT, Carr MJ, et al. Alcohol-related mortality in patients with psoriasis: A population-based cohort study. JAMA Dermatology. 2017;153(12):1256–62. doi: 10.1001/jamadermatol.2017.3225 28914955 PMC5817445

[31] Costentin CE, Mourad A, Lahmek P, Causse X, Pariente A, Hagège H, et al. Hepatocellular carcinoma is diagnosed at a later stage in alcoholic patients: Results of a prospective, nationwide study. Cancer. 2018;124(9):1964–72. doi: 10.1002/cncr.31215 29589878

[32] Venables ZC, Nijsten T, Wong KF, Autier P, Broggio J, Deas A, et al. Epidemiology of basal and cutaneous squamous cell carcinoma in the U.K. 2013–15: a cohort study. Br J Dermatol. 2019;181(3):474–82. doi: 10.1111/bjd.17873 30864158 PMC7379277

[33] Shack L, Jordan C, Thomson CS, et al. Variation in incidence of breast, lung and cervical cancer and malignant melanoma of skin by socioeconomic group in England. BMC Cancer. 2008;8(1):271. doi: 10.1186/1471-2407-8-271 18822122 PMC2577116

[34] Coughlin SS. A review of social determinants of prostate cancer risk, stage, and survival. Prostate Int. 2020;8(2):49–54. doi: 10.1016/j.prnil.2019.08.001 32647640 PMC7335972

