# Supplementary material for: Concordance and timing in recording cancer events in primary care, hospital and mortality records for patients with and without psoriasis: A population-based cohort study
Source: PLoS One. 2021 Jul 19;16(7):e0254661. doi: 10.1371/journal.pone.0254661 (PMC8289076; doi:10.1371/journal.pone.0254661)
Supplement: S1 Fig — (DOCX) [file pone.0254661.s001.docx]

**S1 Fig. Diagram of participant selection process**

Patients eligible for linkage, with a record of psoriasis between 1/1/1998 and 30/11/2018. Excluding those with previous CPRD record of cancer or HIV. N (GOLD) = 59,270 N (Aurum) = 214,700

Patients matched up to 1:6 on age, sex and general practice

Patients eligible for linkage, with no record of psoriasis. Excluding those with previous CPRD record of cancer or HIV. N (GOLD) = 323,759 N (Aurum) = 1,168,734

Patients with previous HES record of cancer N (GOLD) = 365 N (Aurum) = 1,294

Patients with no case N (GOLD) = 1,787 N (Aurum) = 6,286

Patients with previous HES record of cancer N (GOLD) = 1,654 N (Aurum) = 6,051

Patients with no comparison N (GOLD) = 1 N (Aurum) = 3

Patients with death prior to index N (GOLD) = 0 N (Aurum) = 3

Patients with death prior to index N (GOLD) = 94 N (Aurum) = 459

Psoriasis patients N (GOLD) = 58,905 N (Aurum) = 213,406

Comparison patients N (GOLD) = 321,972 N (Aurum) = 1,162,448

Psoriasis patients N (GOLD) = 58,904 N (Aurum) = 213,403

Psoriasis patients N (GOLD) = 58,904 N (Aurum) = 213,400

Comparison patients N (GOLD) = 320,318 N (Aurum) = 1,156,397

Comparison patients N (GOLD) = 320,224 N (Aurum) = 1,155,938
