# Supplementary figures and images for: Concordance and timing in recording cancer events in primary care, hospital and mortality records for patients with and without psoriasis: A population-based cohort study
Source: PLoS One. 2021 Jul 19;16(7):e0254661. doi: 10.1371/journal.pone.0254661 (PMC8289076; doi:10.1371/journal.pone.0254661)

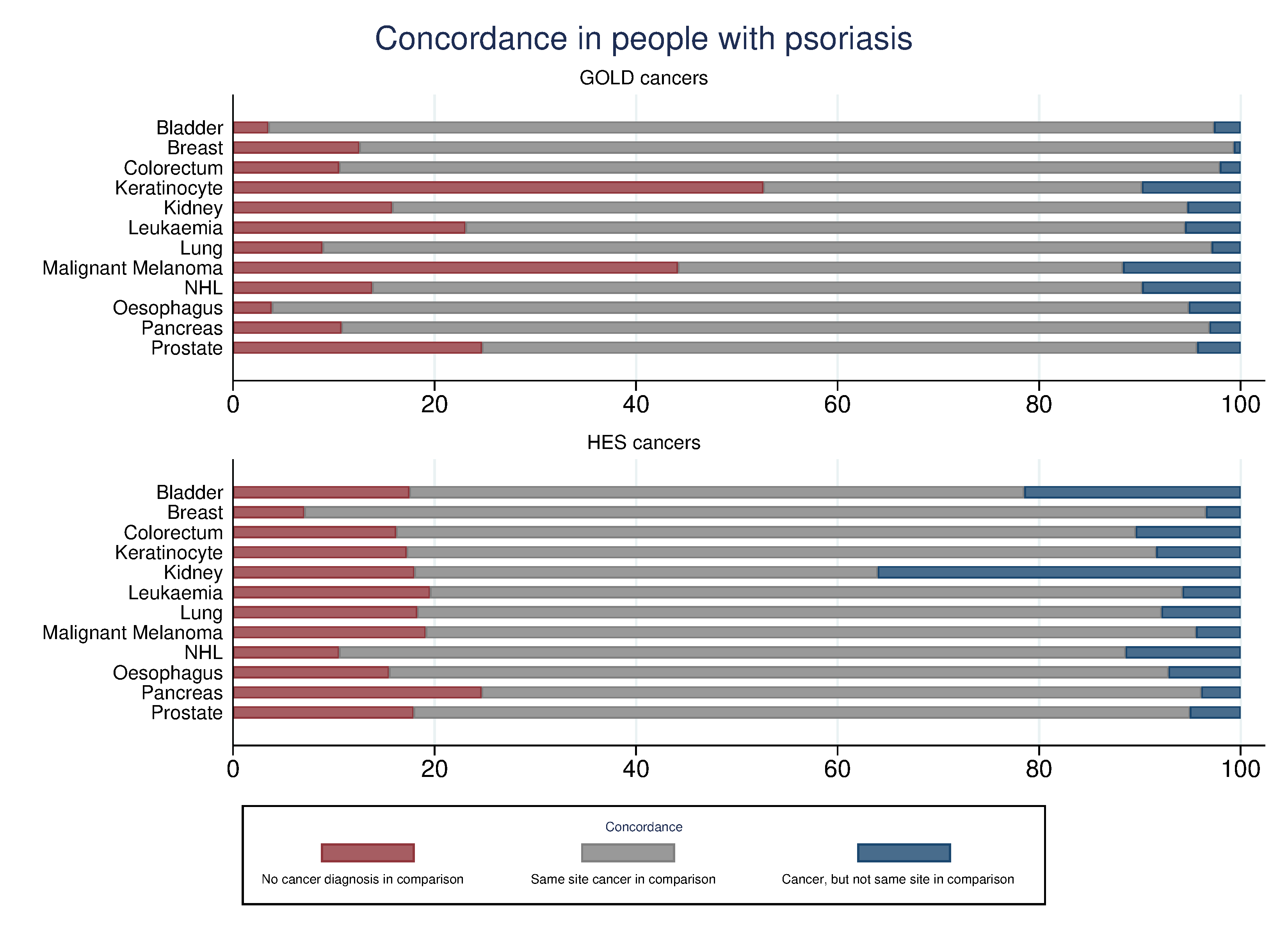

Supplement: S2 Fig — (TIF) [file pone.0254661.s002.tif]

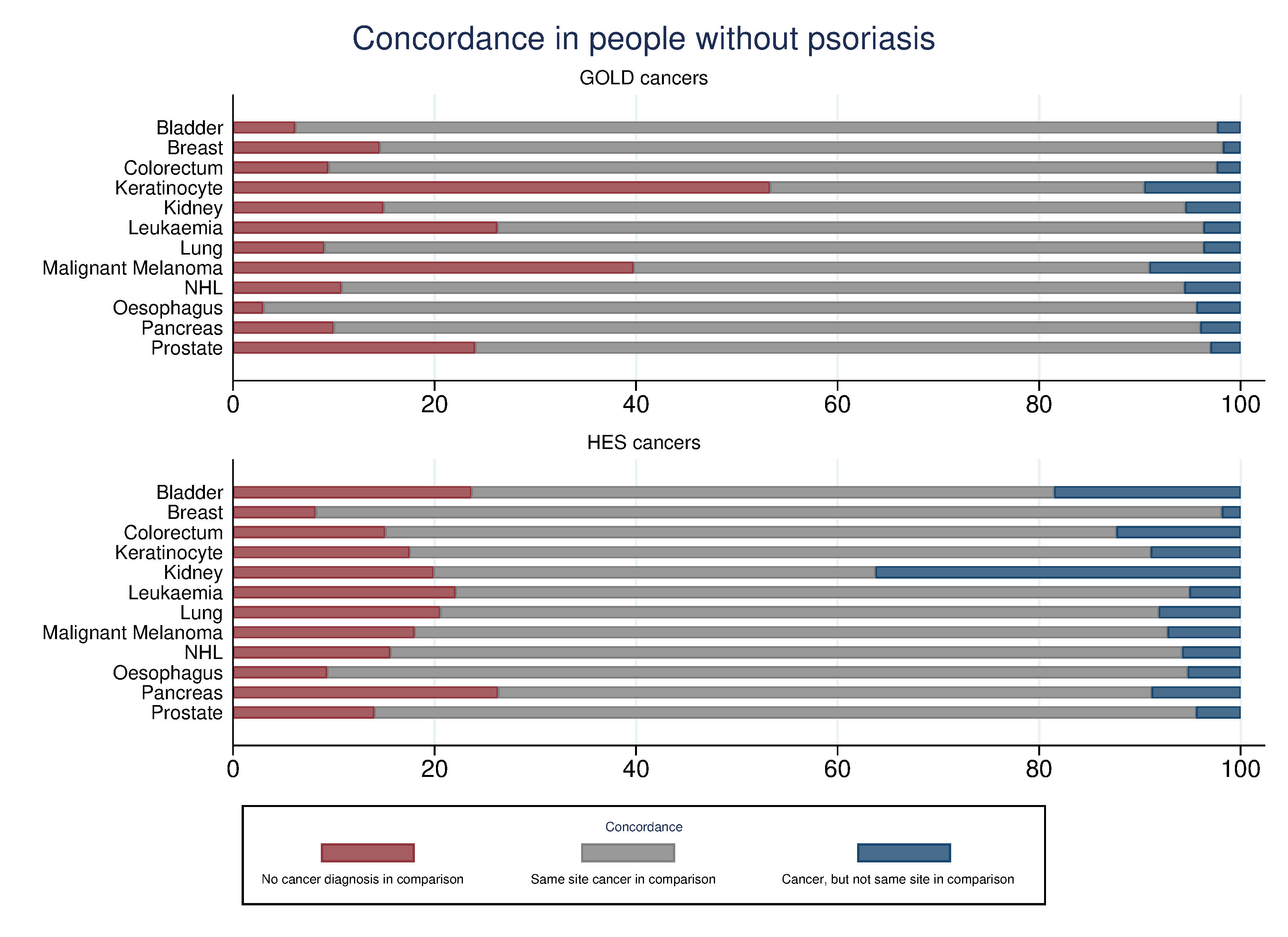

Supplement: S3 Fig — (TIF) [file pone.0254661.s003.tif]

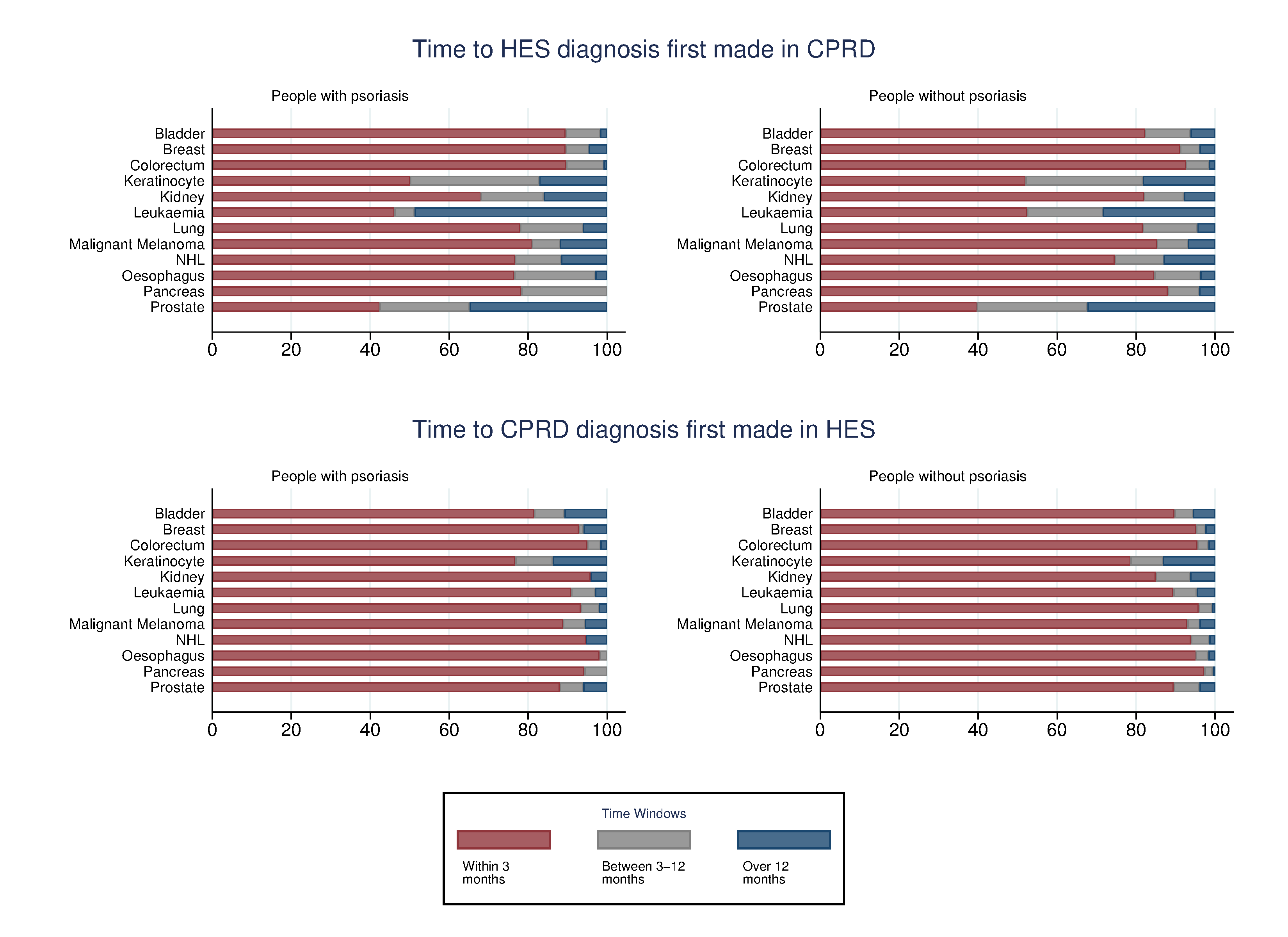

Supplement: S4 Fig — (TIF) [file pone.0254661.s004.tif]
