# Supplementary material for: Concordance and timing in recording cancer events in primary care, hospital and mortality records for patients with and without psoriasis: A population-based cohort study
Source: PLoS One. 2021 Jul 19;16(7):e0254661. doi: 10.1371/journal.pone.0254661 (PMC8289076; doi:10.1371/journal.pone.0254661)
Supplement: S1 Table — (DOCX) [file pone.0254661.s005.docx]

**S1 Table. Concordance in cancer recording for Aurum-linked people with psoriasis**

|  | **Aurum** | | | |  |  |  | **HES** | | | |  |  |
| --- | --- | --- | --- | --- | --- | --- | --- | --- | --- | --- | --- | --- | --- |
| **Site** | **Only Aurum** | **Same site HES** | **Any Site HES** | **Total** |  | **ONS Cancer Death** |  | **Only HES** | **Same site Aurum** | **Any Site Aurum** | **Total** |  | **ONS Cancer Death** |
| **Bladder** | 35 (6.21) | 520 (92.2) | 529 (93.79) | 564 (100) |  | 159 (28.19) |  | 122 (15.78) | 520 (67.27) | 651 (84.21) | 773 (100) |  | 223 (28.85) |
| **Brain** | 17 (10.12) | 140 (83.33) | 151 (89.88) | 168 (100) |  | 108 (64.29) |  | 37 (20.33) | 139 (76.37) | 145 (79.67) | 182 (100) |  | 115 (63.19) |
| **Breast** | 248 (14.34) | 1464 (84.67) | 1481 (85.65) | 1729 (100) |  | 235 (13.59) |  | 97 (6.13) | 1460 (92.29) | 1485 (93.86) | 1582 (100) |  | 227 (14.35) |
| **Cervix** | 12 (13.79) | 62 (71.26) | 75 (86.2) | 87 (100) |  | 27 (31.03) |  | 12 (13.79) | 63 (72.41) | 75 (86.2) | 87 (100) |  | 29 (33.33) |
| **Colorectum** | 78 (6.66) | 1065 (90.87) | 1094 (93.34) | 1172 (100) |  | 442 (37.71) |  | 150 (11.09) | 1055 (77.97) | 1203 (88.91) | 1353 (100) |  | 518 (38.29) |
| **HL** | <5 | 60 (92.31) | 62 (95.38) | 65 (100) |  | 11 (16.92) |  | <5 | 60 (86.96) | 68 (98.55) | 69 (100) |  | 15 (21.74) |
| **Keratinocyte** | 2632 (46.15) | 2508 (43.98) | 3071 (53.84) | 5703 (100) |  | 377 (6.61) |  | 461 (14.08) | 2508 (76.6) | 2813 (85.91) | 3274 (100) |  | 236 (7.21) |
| **Kidney** | 28 (12.5) | 184 (82.14) | 196 (87.5) | 224 (100) |  | 69 (30.8) |  | 66 (19.24) | 184 (53.64) | 277 (80.75) | 343 (100) |  | 116 (33.82) |
| **Larynx** | 8 (9.88) | 67 (82.72) | 73 (90.12) | 81 (100) |  | 23 (28.4) |  | 9 (9.28) | 64 (65.98) | 88 (90.72) | 97 (100) |  | 30 (30.93) |
| **Leukaemia** | 69 (20.29) | 261 (76.76) | 271 (79.7) | 340 (100) |  | 146 (42.94) |  | 55 (16.92) | 256 (78.77) | 270 (83.07) | 325 (100) |  | 169 (52) |
| **Liver** | 23 (12.04) | 151 (79.06) | 168 (87.95) | 191 (100) |  | 128 (67.02) |  | 64 (27.59) | 146 (62.93) | 168 (72.41) | 232 (100) |  | 154 (66.38) |
| **Lung** | 119 (8.3) | 1274 (88.9) | 1314 (91.69) | 1433 (100) |  | 1059 (73.9) |  | 298 (17.89) | 1261 (75.69) | 1368 (82.11) | 1666 (100) |  | 1229 (73.77) |
| **Malignant Melanoma** | 185 (34.07) | 288 (53.04) | 358 (65.93) | 543 (100) |  | 63 (11.6) |  | 56 (15.22) | 284 (77.17) | 312 (84.78) | 368 (100) |  | 52 (14.13) |
| **Multiple Myeloma** | 27 (16.27) | 134 (80.72) | 139 (83.73) | 166 (100) |  | 61 (36.75) |  | 33 (18.86) | 137 (78.29) | 142 (81.14) | 175 (100) |  | 67 (38.29) |
| **NHL** | 74 (15.71) | 370 (78.56) | 397 (84.28) | 471 (100) |  | 154 (32.7) |  | 63 (13.76) | 361 (78.82) | 395 (86.24) | 458 (100) |  | 182 (39.74) |
| **Oesophagus** | 8 (2.27) | 330 (93.48) | 345 (97.73) | 353 (100) |  | 251 (71.1) |  | 38 (10.08) | 318 (84.35) | 339 (89.92) | 377 (100) |  | 259 (68.7) |
| **Oral Cavity** | 8 (8.99) | 70 (78.65) | 81 (91.01) | 89 (100) |  | 30 (33.71) |  | 26 (14.53) | 70 (39.11) | 153 (85.47) | 179 (100) |  | 58 (32.4) |
| **Ovary** | 25 (11.96) | 144 (68.9) | 184 (88.03) | 209 (100) |  | 88 (42.11) |  | 44 (22) | 139 (69.5) | 156 (78) | 200 (100) |  | 104 (52) |
| **Pancreas** | 25 (10.12) | 207 (83.81) | 222 (89.87) | 247 (100) |  | 206 (83.4) |  | 81 (25.47) | 207 (65.09) | 237 (74.52) | 318 (100) |  | 257 (80.82) |
| **Prostate** | 268 (17.43) | 1235 (80.3) | 1270 (82.57) | 1538 (100) |  | 304 (19.77) |  | 135 (9.64) | 1222 (87.29) | 1265 (90.35) | 1400 (100) |  | 296 (21.14) |
| **Stomach** | 9 (4.89) | 153 (83.15) | 175 (95.1) | 184 (100) |  | 121 (65.76) |  | 37 (16.09) | 147 (63.91) | 193 (83.91) | 230 (100) |  | 158 (68.7) |
| **Thyroid** | 18 (25.71) | 50 (71.43) | 52 (74.28) | 70 (100) |  | 11 (15.71) |  | 14 (20) | 49 (70) | 56 (80) | 70 (100) |  | 8 (11.43) |
| **Uterus** | 20 (10.1) | 172 (86.87) | 178 (89.89) | 198 (100) |  | 31 (15.66) |  | 35 (12.77) | 179 (65.33) | 239 (87.22) | 274 (100) |  | 54 (19.71) |
| **Any Cancer (exc keratinocyte)** | 1837 (15.45) | 8438 (70.97) | 10052 (84.54) | 11889 (100) |  | 4189 (35.23) |  | 1881 (15.97) | 8353 (70.93) | 9896 (84.02) | 11777 (100) |  | 4789 (40.66) |
