# Supplementary material for: Concordance and timing in recording cancer events in primary care, hospital and mortality records for patients with and without psoriasis: A population-based cohort study
Source: PLoS One. 2021 Jul 19;16(7):e0254661. doi: 10.1371/journal.pone.0254661 (PMC8289076; doi:10.1371/journal.pone.0254661)
Supplement: S2 Table — (DOCX) [file pone.0254661.s006.docx]

**S2 Table. Concordance in cancer recording for Aurum-linked comparison patients**

|  | **Aurum** | | | |  |  |  | **HES** | | | |  |  |
| --- | --- | --- | --- | --- | --- | --- | --- | --- | --- | --- | --- | --- | --- |
| **Site** | **Only Aurum** | **Same site HES** | **Any Site HES** | **Total** |  | **ONS Cancer Death** |  | **Only HES** | **Same site Aurum** | **Any Site Aurum** | **Total** |  | **ONS Cancer Death** |
| **Bladder** | 142 (5.25) | 2522 (93.3) | 2561 (94.74) | 2703 (100) |  | 722 (26.71) |  | 663 (17.11) | 2531 (65.33) | 3211 (82.88) | 3874 (100) |  | 1072 (27.67) |
| **Brain** | 130 (13.44) | 775 (80.14) | 837 (86.55) | 967 (100) |  | 610 (63.08) |  | 190 (19.23) | 770 (77.94) | 798 (80.76) | 988 (100) |  | 611 (61.84) |
| **Breast** | 1367 (13.79) | 8425 (84.98) | 8547 (86.21) | 9914 (100) |  | 1406 (14.18) |  | 674 (7.31) | 8396 (91.11) | 8541 (92.68) | 9215 (100) |  | 1407 (15.27) |
| **Cervix** | 50 (11.63) | 346 (80.47) | 380 (88.37) | 430 (100) |  | 105 (24.42) |  | 57 (12.53) | 340 (74.73) | 398 (87.47) | 455 (100) |  | 127 (27.91) |
| **Colorectum** | 435 (6.76) | 5862 (91.08) | 6001 (93.24) | 6436 (100) |  | 2422 (37.63) |  | 958 (12.38) | 5863 (75.79) | 6778 (87.61) | 7736 (100) |  | 3014 (38.96) |
| **HL** | 9 (4.95) | 162 (89.01) | 173 (95.05) | 182 (100) |  | 37 (20.33) |  | 15 (7.81) | 150 (78.12) | 177 (92.18) | 192 (100) |  | 43 (22.4) |
| **Keratinocyte** | 13911 (45.88) | 13478 (44.45) | 16409 (54.11) | 30320 (100) |  | 1776 (5.86) |  | 2395 (13.75) | 13478 (77.39) | 15021 (86.24) | 17416 (100) |  | 1215 (6.98) |
| **Kidney** | 150 (12.91) | 959 (82.53) | 1012 (87.09) | 1162 (100) |  | 413 (35.54) |  | 331 (19.61) | 957 (56.69) | 1357 (80.39) | 1688 (100) |  | 634 (37.56) |
| **Larynx** | 23 (7.19) | 251 (78.44) | 297 (92.81) | 320 (100) |  | 108 (33.75) |  | 62 (14.25) | 243 (55.86) | 373 (85.74) | 435 (100) |  | 159 (36.55) |
| **Leukaemia** | 406 (22.92) | 1293 (73.01) | 1365 (77.07) | 1771 (100) |  | 656 (37.04) |  | 274 (17.1) | 1260 (78.65) | 1328 (82.89) | 1602 (100) |  | 777 (48.5) |
| **Liver** | 93 (11.34) | 621 (75.73) | 727 (88.65) | 820 (100) |  | 597 (72.8) |  | 186 (19.7) | 624 (66.1) | 758 (80.29) | 944 (100) |  | 670 (70.97) |
| **Lung** | 511 (7.9) | 5731 (88.62) | 5956 (92.09) | 6467 (100) |  | 4797 (74.18) |  | 1534 (19.83) | 5679 (73.43) | 6200 (80.16) | 7734 (100) |  | 5723 (74) |
| **Malignant Melanoma** | 1012 (32.34) | 1796 (57.4) | 2117 (67.65) | 3129 (100) |  | 350 (11.19) |  | 283 (12.67) | 1792 (80.21) | 1951 (87.33) | 2234 (100) |  | 294 (13.16) |
| **Multiple Myeloma** | 79 (8.49) | 835 (89.78) | 851 (91.5) | 930 (100) |  | 411 (44.19) |  | 148 (14.48) | 830 (81.21) | 874 (85.51) | 1022 (100) |  | 461 (45.11) |
| **NHL** | 237 (10.2) | 1973 (84.9) | 2087 (89.8) | 2324 (100) |  | 710 (30.55) |  | 256 (10.77) | 1954 (82.17) | 2122 (89.23) | 2378 (100) |  | 852 (35.83) |
| **Oesophagus** | 56 (3.12) | 1651 (91.98) | 1739 (96.88) | 1795 (100) |  | 1324 (73.76) |  | 182 (9.87) | 1531 (83.03) | 1662 (90.13) | 1844 (100) |  | 1337 (72.51) |
| **Oral Cavity** | 49 (9.9) | 395 (79.8) | 446 (90.1) | 495 (100) |  | 169 (34.14) |  | 103 (12.32) | 387 (46.29) | 733 (87.67) | 836 (100) |  | 271 (32.42) |
| **Ovary** | 133 (10.45) | 902 (70.86) | 1140 (89.55) | 1273 (100) |  | 628 (49.33) |  | 275 (21.11) | 880 (67.54) | 1028 (78.89) | 1303 (100) |  | 731 (56.1) |
| **Pancreas** | 119 (8.49) | 1178 (84.08) | 1282 (91.5) | 1401 (100) |  | 1158 (82.66) |  | 416 (24.21) | 1167 (67.93) | 1302 (75.78) | 1718 (100) |  | 1404 (81.72) |
| **Prostate** | 1779 (18.67) | 7550 (79.22) | 7752 (81.33) | 9531 (100) |  | 1842 (19.33) |  | 861 (10.15) | 7406 (87.3) | 7622 (89.85) | 8483 (100) |  | 1851 (21.82) |
| **Stomach** | 53 (5.38) | 820 (83.16) | 933 (94.62) | 986 (100) |  | 680 (68.97) |  | 206 (15.61) | 799 (60.53) | 1114 (84.39) | 1320 (100) |  | 952 (72.12) |
| **Thyroid** | 98 (24.02) | 298 (73.04) | 310 (75.98) | 408 (100) |  | 39 (9.56) |  | 94 (21.66) | 302 (69.59) | 340 (78.34) | 434 (100) |  | 57 (13.13) |
| **Uterus** | 112 (9.26) | 1046 (86.45) | 1098 (90.74) | 1210 (100) |  | 258 (21.32) |  | 192 (12.14) | 1080 (68.27) | 1390 (87.86) | 1582 (100) |  | 357 (22.57) |
| **Any Cancer (exc keratinocyte)** | 9706 (15.24) | 45547 (71.51) | 53985 (84.76) | 63691 (100) |  | 22002 (34.54) |  | 9988 (15.78) | 45085 (71.23) | 53310 (84.22) | 63298 (100) |  | 25384 (40.1) |
