# Supplementary material for: Concordance and timing in recording cancer events in primary care, hospital and mortality records for patients with and without psoriasis: A population-based cohort study
Source: PLoS One. 2021 Jul 19;16(7):e0254661. doi: 10.1371/journal.pone.0254661 (PMC8289076; doi:10.1371/journal.pone.0254661)
Supplement: S3 Table — (DOCX) [file pone.0254661.s007.docx]

**S3 Table. Concordance in cancer recording for GOLD-linked people with psoriasis**

|  | **CPRD GOLD** | | | | | |  | **HES** | | | |  |  |
| --- | --- | --- | --- | --- | --- | --- | --- | --- | --- | --- | --- | --- | --- |
| **Site** | **Only GOLD** | **Same site in HES** | **Any Site in HES** | **Total** |  | **ONS Cancer Death** |  | **Only HES** | **Same site in GOLD** | **Any Site in GOLD** | **Total** |  | **ONS Cancer Death** |
| **Bladder** | <5 | 107 (93.86) | 110 (96.49) | 114 (100) |  | 34 (29.82) |  | 31 (17.51) | 108 (61.02) | 146 (82.48) | 177 (100) |  | 51 (28.81) |
| **Brain** | <5 | 35 (85.37) | 38 (92.68) | 41 (100) |  | 25 (60.98) |  | 6 (14.29) | 35 (83.33) | 36 (85.71) | 42 (100) |  | 24 (57.14) |
| **Breast** | 57 (12.53) | 395 (86.81) | 398 (87.47) | 455 (100) |  | 67 (14.73) |  | 31 (7.08) | 392 (89.5) | 407 (92.92) | 438 (100) |  | 69 (15.75) |
| **Cervix** | <5 | 16 (80) | 17 (85) | 20 (100) |  | <5 |  | <5 | 15 (83.33) | 15 (83.33) | 18 (100) |  | <5 |
| **Colorectum** | 31 (10.54) | 257 (87.41) | 263 (89.45) | 294 (100) |  | 96 (32.65) |  | 56 (16.18) | 254 (73.41) | 290 (83.81) | 346 (100) |  | 125 (36.13) |
| **HL** | <5 | 11 (84.62) | 11 (84.61) | 13 (100) |  | <5 |  | <5 | 11 (73.33) | 13 (86.66) | 15 (100) |  | 6 (40) |
| **Keratinocyte** | 786 (52.65) | 561 (37.58) | 707 (47.35) | 1493 (100) |  | 86 (5.76) |  | 130 (17.24) | 561 (74.4) | 624 (82.75) | 754 (100) |  | 47 (6.23) |
| **Kidney** | 9 (15.79) | 45 (78.95) | 48 (84.21) | 57 (100) |  | 22 (38.6) |  | 18 (18) | 46 (46) | 82 (82) | 100 (100) |  | 37 (37) |
| **Larynx** | <5 | 11 (73.33) | 14 (93.33) | 15 (100) |  | <5 |  | <5 | 10 (58.82) | 16 (94.11) | 17 (100) |  | <5 |
| **Leukaemia** | 21 (23.08) | 65 (71.43) | 70 (76.92) | 91 (100) |  | 39 (42.86) |  | 17 (19.54) | 65 (74.71) | 70 (80.45) | 87 (100) |  | 43 (49.43) |
| **Liver** | 8 (21.62) | 25 (67.57) | 29 (78.37) | 37 (100) |  | 23 (62.16) |  | 14 (32.56) | 24 (55.81) | 29 (67.44) | 43 (100) |  | 31 (72.09) |
| **Lung** | 31 (8.88) | 308 (88.25) | 318 (91.11) | 349 (100) |  | 264 (75.64) |  | 77 (18.29) | 311 (73.87) | 344 (81.71) | 421 (100) |  | 324 (76.96) |
| **Malignant Melanoma** | 53 (44.17) | 53 (44.17) | 67 (55.83) | 120 (100) |  | 13 (10.83) |  | 13 (19.12) | 52 (76.47) | 55 (80.88) | 68 (100) |  | 9 (13.24) |
| **Multiple Myeloma** | 6 (14.29) | 36 (85.71) | 36 (85.71) | 42 (100) |  | 15 (35.71) |  | 10 (19.23) | 38 (73.08) | 42 (80.76) | 52 (100) |  | 18 (34.62) |
| **NHL** | 17 (13.82) | 94 (76.42) | 106 (86.17) | 123 (100) |  | 37 (30.08) |  | 12 (10.53) | 89 (78.07) | 102 (89.47) | 114 (100) |  | 41 (35.96) |
| **Oesophagus** | <5 | 71 (91.03) | 75 (96.15) | 78 (100) |  | 57 (73.08) |  | 13 (15.48) | 65 (77.38) | 71 (84.52) | 84 (100) |  | 59 (70.24) |
| **Oral Cavity** | <5 | 20 (86.96) | 20 (86.95) | 23 (100) |  | 6 (26.09) |  | 5 (11.11) | 19 (42.22) | 40 (88.88) | 45 (100) |  | 12 (26.67) |
| **Ovary** | 9 (18) | 32 (64) | 41 (82) | 50 (100) |  | 25 (50) |  | 17 (32.08) | 31 (58.49) | 36 (67.92) | 53 (100) |  | 28 (52.83) |
| **Pancreas** | 7 (10.77) | 56 (86.15) | 58 (89.23) | 65 (100) |  | 54 (83.08) |  | 19 (24.68) | 55 (71.43) | 58 (75.32) | 77 (100) |  | 60 (77.92) |
| **Prostate** | 86 (24.71) | 247 (70.98) | 262 (75.28) | 348 (100) |  | 66 (18.97) |  | 57 (17.92) | 245 (77.04) | 261 (82.07) | 318 (100) |  | 69 (21.7) |
| **Stomach** | <5 | 39 (82.98) | 45 (95.74) | 47 (100) |  | 34 (72.34) |  | 10 (14.93) | 40 (59.7) | 57 (85.07) | 67 (100) |  | 45 (67.16) |
| **Thyroid** | 5 (29.41) | 9 (52.94) | 12 (70.58) | 17 (100) |  | <5 |  | 5 (27.78) | 9 (50) | 13 (72.22) | 18 (100) |  | <5 |
| **Uterus** | 5 (11.11) | 39 (86.67) | 40 (88.88) | 45 (100) |  | 8 (17.78) |  | 7 (12.28) | 38 (66.67) | 50 (87.71) | 57 (100) |  | 11 (19.3) |
| **Any cancer (Exc keratinocyte)** | 504 (17.28) | 1979 (67.87) | 2412 (82.71) | 2916 (100) |  | 1029 (35.28) |  | 537 (18.45) | 1959 (67.3) | 2374 (81.55) | 2911 (100) |  | 1188 (40.81) |
