# Supplementary material for: Concordance and timing in recording cancer events in primary care, hospital and mortality records for patients with and without psoriasis: A population-based cohort study
Source: PLoS One. 2021 Jul 19;16(7):e0254661. doi: 10.1371/journal.pone.0254661 (PMC8289076; doi:10.1371/journal.pone.0254661)
Supplement: S4 Table — (DOCX) [file pone.0254661.s008.docx]

**S4 Table. Concordance in cancer recording for GOLD-linked comparison patients**

|  | **GOLD** | | | |  |  |  | **HES** | | | |  |  |
| --- | --- | --- | --- | --- | --- | --- | --- | --- | --- | --- | --- | --- | --- |
| **Site** | **Only GOLD** | **Same site in HES** | **Any Site in HES** | **Total** |  | **ONS Cancer Death** |  | **Only HES** | **Same site in GOLD** | **Any Site in GOLD** | **Total** |  | **ONS Cancer Death** |
| **Bladder** | 37 (6.16) | 550 (91.51) | 564 (93.84) | 601 (100) |  | 174 (28.95) |  | 220 (23.66) | 538 (57.85) | 710 (76.34) | 930 (100) |  | 249 (26.77) |
| **Brain** | 36 (16.59) | 160 (73.73) | 181 (83.41) | 217 (100) |  | 127 (58.53) |  | 66 (27.62) | 160 (66.95) | 173 (72.38) | 239 (100) |  | 130 (54.39) |
| **Breast** | 351 (14.55) | 2020 (83.71) | 2062 (85.45) | 2413 (100) |  | 320 (13.26) |  | 184 (8.19) | 2020 (89.94) | 2062 (91.8) | 2246 (100) |  | 311 (13.85) |
| **Cervix** | 11 (11.11) | 80 (80.81) | 88 (88.88) | 99 (100) |  | 29 (29.29) |  | 12 (11.32) | 80 (75.47) | 94 (88.67) | 106 (100) |  | 33 (31.13) |
| **Colorectum** | 148 (9.46) | 1379 (88.17) | 1416 (90.53) | 1564 (100) |  | 530 (33.89) |  | 288 (15.09) | 1385 (72.59) | 1620 (84.9) | 1908 (100) |  | 701 (36.74) |
| **HL** | <5 | 45 (86.54) | 50 (96.15) | 52 (100) |  | 8 (15.38) |  | 5 (9.62) | 42 (80.77) | 47 (90.38) | 52 (100) |  | 8 (15.38) |
| **Keratinocyte** | 4120 (53.28) | 2875 (37.18) | 3613 (46.72) | 7733 (100) |  | 433 (5.6) |  | 684 (17.51) | 2875 (73.59) | 3223 (82.49) | 3907 (100) |  | 273 (6.99) |
| **Kidney** | 30 (14.93) | 160 (79.6) | 171 (85.07) | 201 (100) |  | 76 (37.81) |  | 73 (19.89) | 161 (43.87) | 294 (80.1) | 367 (100) |  | 142 (38.69) |
| **Larynx** | 9 (14.75) | 50 (81.97) | 52 (85.24) | 61 (100) |  | 18 (29.51) |  | 21 (20) | 49 (46.67) | 84 (80) | 105 (100) |  | 29 (27.62) |
| **Leukaemia** | 107 (26.23) | 286 (70.1) | 301 (73.77) | 408 (100) |  | 163 (39.95) |  | 87 (22.08) | 287 (72.84) | 307 (77.91) | 394 (100) |  | 195 (49.49) |
| **Liver** | 26 (14.53) | 125 (69.83) | 153 (85.47) | 179 (100) |  | 124 (69.27) |  | 41 (21.35) | 121 (63.02) | 151 (78.64) | 192 (100) |  | 133 (69.27) |
| **Lung** | 133 (9.04) | 1284 (87.29) | 1338 (90.95) | 1471 (100) |  | 1144 (77.77) |  | 372 (20.55) | 1291 (71.33) | 1438 (79.44) | 1810 (100) |  | 1367 (75.52) |
| **Malignant Melanoma** | 281 (39.75) | 362 (51.2) | 426 (60.25) | 707 (100) |  | 80 (11.32) |  | 87 (18.01) | 361 (74.74) | 396 (81.98) | 483 (100) |  | 81 (16.77) |
| **Multiple Myeloma** | 25 (10.42) | 213 (88.75) | 215 (89.58) | 240 (100) |  | 95 (39.58) |  | 45 (17.05) | 210 (79.55) | 219 (82.95) | 264 (100) |  | 107 (40.53) |
| **NHL** | 58 (10.76) | 451 (83.67) | 481 (89.23) | 539 (100) |  | 161 (29.87) |  | 89 (15.61) | 448 (78.6) | 481 (84.38) | 570 (100) |  | 204 (35.79) |
| **Oesophagus** | 13 (3) | 402 (92.63) | 421 (97) | 434 (100) |  | 322 (74.19) |  | 41 (9.34) | 375 (85.42) | 398 (90.66) | 439 (100) |  | 315 (71.75) |
| **Oral Cavity** | 13 (10.83) | 92 (76.67) | 107 (89.16) | 120 (100) |  | 38 (31.67) |  | 43 (20.98) | 86 (41.95) | 162 (79.02) | 205 (100) |  | 47 (22.93) |
| **Ovary** | 37 (13.41) | 201 (72.83) | 239 (86.59) | 276 (100) |  | 137 (49.64) |  | 65 (21.17) | 200 (65.15) | 242 (78.82) | 307 (100) |  | 168 (54.72) |
| **Pancreas** | 35 (9.97) | 302 (86.04) | 316 (90.02) | 351 (100) |  | 299 (85.19) |  | 122 (26.29) | 301 (64.87) | 342 (73.7) | 464 (100) |  | 365 (78.66) |
| **Prostate** | 524 (24.03) | 1592 (72.99) | 1657 (75.97) | 2181 (100) |  | 361 (16.55) |  | 270 (14.03) | 1570 (81.56) | 1655 (85.97) | 1925 (100) |  | 388 (20.16) |
| **Stomach** | 20 (8.77) | 189 (82.89) | 208 (91.22) | 228 (100) |  | 155 (67.98) |  | 55 (17.13) | 190 (59.19) | 266 (82.86) | 321 (100) |  | 235 (73.21) |
| **Thyroid** | 34 (32.69) | 67 (64.42) | 70 (67.3) | 104 (100) |  | 9 (8.65) |  | 22 (19.64) | 67 (59.82) | 90 (80.35) | 112 (100) |  | 15 (13.39) |
| **Uterus** | 33 (10.25) | 275 (85.4) | 289 (89.75) | 322 (100) |  | 78 (24.22) |  | 54 (13.6) | 276 (69.52) | 343 (86.39) | 397 (100) |  | 91 (22.92) |
| **Any cancer (Exc keratinocyte)** | 2712 (17.8) | 10319 (67.73) | 12524 (82.2) | 15236 (100) |  | 5145 (33.76) |  | 2779 (18.37) | 10250 (67.77) | 12345 (81.62) | 15124 (100) |  | 5985 (39.57) |
