# Supplementary material for: Concordance and timing in recording cancer events in primary care, hospital and mortality records for patients with and without psoriasis: A population-based cohort study
Source: PLoS One. 2021 Jul 19;16(7):e0254661. doi: 10.1371/journal.pone.0254661 (PMC8289076; doi:10.1371/journal.pone.0254661)
Supplement: S5 Table — (DOCX) [file pone.0254661.s009.docx]

**S5 Table. Multivariable-adjusted odds of non-concordance according to covariates in CPRD-GOLD linked data**

|  | Psoriasis - GOLD source | | |  | Psoriasis - HES source | | |  | Comparison - GOLD source | | |  | Comparison - HES source | | |
| --- | --- | --- | --- | --- | --- | --- | --- | --- | --- | --- | --- | --- | --- | --- | --- |
|  |  |  |  |  |  |  |  |  |  |  |  |  |  |  |  |
|  | Odds Ratio | 95% CI | |  | Odds Ratio | 95% CI | |  | Odds Ratio | 95% CI | |  | Odds Ratio | 95% CI | |
|  |  |  |  |  |  |  |  |  |  |  |  |  |  |  |  |
| Age categories |  |  |  |  |  |  |  |  |  |  |  |  |  |  |  |
| Under 64 | 1.00 | reference | |  | 1.00 | reference | |  | 1.00 | reference | |  | 1.00 | reference | |
| 65 to 74 | 1.03 | 0.82 | 1.29 |  | 1.26 | 1.01 | 1.58 |  | 0.91 | 0.82 | 1.00 |  | 1.28 | 1.16 | 1.41 |
| Over 75 | 1.49 | 1.16 | 1.91 |  | 2.16 | 1.70 | 2.73 |  | 1.34 | 1.20 | 1.49 |  | 2.11 | 1.90 | 2.34 |
|  |  |  |  |  |  |  |  |  |  |  |  |  |  |  |  |
| Gender |  |  |  |  |  |  |  |  |  |  |  |  |  |  |  |
| Male | 1.00 | reference | |  | 1.00 | reference | |  | 1.00 | reference | |  | 1.00 | reference | |
| Female | 0.94 | 0.77 | 1.14 |  | 0.90 | 0.75 | 1.09 |  | 0.93 | 0.85 | 1.01 |  | 0.91 | 0.84 | 0.99 |
|  |  |  |  |  |  |  |  |  |  |  |  |  |  |  |  |
| Deprivation |  |  |  |  |  |  |  |  |  |  |  |  |  |  |  |
| 1 (least deprived) | 1.00 | reference | |  | 1.00 | reference | |  | 1.00 | reference | |  | 1.00 | reference | |
| 2 | 1.01 | 0.77 | 1.33 |  | 1.00 | 0.76 | 1.31 |  | 0.89 | 0.79 | 0.99 |  | 1.01 | 0.89 | 1.14 |
| 3 | 1.14 | 0.87 | 1.50 |  | 0.96 | 0.72 | 1.28 |  | 0.88 | 0.78 | 0.99 |  | 0.98 | 0.87 | 1.12 |
| 4 | 0.75 | 0.55 | 1.03 |  | 0.93 | 0.68 | 1.25 |  | 0.74 | 0.65 | 0.85 |  | 1.07 | 0.94 | 1.22 |
| 5 (most deprived) | 0.76 | 0.54 | 1.07 |  | 1.11 | 0.81 | 1.51 |  | 0.61 | 0.53 | 0.71 |  | 1.19 | 1.04 | 1.36 |
|  |  |  |  |  |  |  |  |  |  |  |  |  |  |  |  |
| Period |  |  |  |  |  |  |  |  |  |  |  |  |  |  |  |
| 1998/2000 | 1.00 | reference | |  | 1.00 | reference | |  | 1.00 | reference | |  | 1.00 | reference | |
| 2001/2003 | 0.55 | 0.27 | 1.12 |  | 1.01 | 0.45 | 2.28 |  | 0.69 | 0.51 | 0.94 |  | 0.79 | 0.59 | 1.05 |
| 2004/2006 | 0.48 | 0.25 | 0.94 |  | 1.14 | 0.53 | 2.46 |  | 0.68 | 0.51 | 0.91 |  | 0.68 | 0.52 | 0.89 |
| 2007/2009 | 0.64 | 0.33 | 1.21 |  | 1.19 | 0.56 | 2.54 |  | 0.66 | 0.50 | 0.87 |  | 0.57 | 0.43 | 0.74 |
| 2010/2012 | 0.54 | 0.29 | 1.03 |  | 0.90 | 0.42 | 1.93 |  | 0.66 | 0.50 | 0.87 |  | 0.56 | 0.43 | 0.73 |
| 2013/2015 | 0.69 | 0.36 | 1.31 |  | 1.14 | 0.53 | 2.44 |  | 0.74 | 0.56 | 0.98 |  | 0.63 | 0.48 | 0.81 |
| 2016/2018 | 0.90 | 0.47 | 1.74 |  | 1.25 | 0.57 | 2.72 |  | 0.78 | 0.58 | 1.03 |  | 0.84 | 0.64 | 1.10 |
